# Essential HDRescue: A Co-Targeting Strategy to Enhance Precision Genome Editing by Co-Editing Essential Genes

**DOI:** 10.3390/cells15090768

**Published:** 2026-04-24

**Authors:** Jamaica F. Siwak, Jon P. Connelly, Shondra M. Pruett-Miller

**Affiliations:** 1 Center for Advanced Genome Engineering, St. Jude Children’s Research Hospital, Memphis, TN 38103, USA; jamaica.siwak@stjude.org (J.F.S.); patrick.connelly@stjude.org (J.P.C.); 2 Department of Cell and Molecular Biology, St. Jude Children’s Research Hospital, Memphis, TN 38103, USA; 3 Graduate School of Biomedical Sciences, St. Jude Children’s Research Hospital, Memphis, TN 38103, USA; 4 Comprehensive Cancer Center, St. Jude Children’s Research Hospital, Memphis, TN 38103, USA

**Keywords:** HDR, co-targeting, cell line development, CRISPR, essential genes, genome engineering

## Abstract

Genome editing is widely used and conceptually simple, yet in practice, it is hindered by laborious workflows and high costs. These challenges stem from the difficulty of identifying and isolating cells that contain the desired user-defined modifications, a problem compounded by the wide variability in editing efficiencies across cell types. While homology-directed repair (HDR) provides a mechanism for precise genome modification following nuclease-induced double-strand breaks (DSBs), it is frequently outcompeted by the dominant mutagenic non-homologous end-joining (NHEJ) pathway in mammalian cells. Therefore, we developed a novel enrichment method, Essential HDRescue, to increase the frequency of HDR events at a target site by co-targeting an essential genomic locus. Using both intrinsic positive and negative selection at a common essential gene, we enabled enrichment of precise editing events at a second, unlinked target site. We demonstrated that co-targeting essential genes in cancer cell lines and iPSCs increased HDR rates without the need for an exogenous reporter or selective drug. Analysis of resulting clones revealed that Essential HDRescue produced up to a 6-fold increase in single-allele edits and an ~4-fold increase in homozygous edits relative to single-targeted controls. By harnessing the intrinsic cellular dependencies that arise from DSB repair at essential loci, Essential HDRescue offers a widely applicable method to improve precise genome editing outcomes in mammalian cells, leaving only a minimal, protein-silent scar at the essential gene.

## 1. Introduction

CRISPR technologies play a crucial role in many biological applications, including cell modeling, gene and cell therapeutics, and functional genomics [1,2]. CRISPR can be used to engineer mutations at a target site by introducing programmed double-stranded breaks (DSBs) into the genome, which are generally repaired by one of two predominant DNA repair pathways [3]. The first and most active pathway in mammalian cells, non-homologous end joining (NHEJ), is considered error-prone because it frequently introduces insertion or deletion (indel) mutations and is commonly used to generate gene knockouts (KOs) [4]. In contrast, the second pathway, homology-directed repair (HDR), can install precise, user-defined mutations, such as gene fragment insertions or single-nucleotide variants, at a target gene or locus of interest using a homologous DNA template [4,5,6]. However, HDR occurs less often than NHEJ in somatic mammalian cells, making the development of engineered cells for both basic and preclinical research more labor intensive and costly. In the absence of a reporter, HDR-edited cells typically appear phenotypically indistinguishable from NHEJ- or non-edited cells, making targeted cell identification challenging and further limiting the practical application of genome engineering [7]. Numerous strategies can enhance HDR by using small molecules to either suppress NHEJ activity or arrest cells in G2/M phase, when homologous recombination genes are expressed [8]. However, some of these drugs cause chromosomal instability, necessitating further investigation of aberrant DSB repair by a site-directed nuclease [9]. Newer genome editing tools, such as base editors and prime editors avoid generating DSBs, offer alternatives to HDR- and NHEJ-based editing altogether [10,11,12]. However, each technique has its own limitations. Base editors are restricted to making specific point mutations and can introduce unwanted bystander edits when nearby editable bases fall within the editing window [13]. Prime editors offer greater flexibility than base editors but often have low efficiencies across many cell types and require extensive optimization of prime editing guide RNA design, nicking strategies, and delivery conditions [14]. Such constraints limit the broad applicability of these newer technologies despite their potential advantages. Given the potential impact of CRISPR technologies across biological fields, it is vital to continue developing novel approaches that produce precisely edited cells while improving upon current efficiency rates.

A variety of selection strategies have been developed to increase the representation of edited cells within a larger population [15]. The most common and conceptually straightforward approach is to directly couple the desired edit to a genetic reporter, such as a fluorescent protein or antibiotic-resistance gene, enabling edited cells to be readily distinguished from unedited ones [16,17,18]. This approach generally introduces a transgene, which is not always desirable and can limit the number of possible edits if available selection markers are exhausted. In contrast, co-targeting represents a conceptually distinct strategy in which a gain-of-function mutation at one locus serves as the selectable event, thereby enriching for phenotypically silent edits at a second, unlinked site. Because editing outcomes at two independent loci are not statistically independent, simultaneous targeting can enrich for double-editing events [19,20,21,22]. Such co-targeting strategies have been adopted in model organisms (e.g., *Caenorhabditis elegans* and *Drosophila*), as well as in mammalian cells [23,24,25,26,27]. Some co-targeting strategies use cytotoxic agents, such as drugs, toxins, or antibiotics, to impose positive or negative selection at the reporter locus [21,28,29,30,31,32]. Though effective, these strategies can introduce non-natural elements at the reporter locus, which may have negative consequences on downstream applications, particularly if naïve cells are required for pre-clinical research. Other strategies require physical separation of cells using specialized equipment, such as fluorescence-activated cell sorting or magnetic separation [29,33,34]. Still other strategies require the insertion of whole transgenes into an exon of a gene required for cell viability [35]. This restricts the knock-in (KI) to a single copy DNA, which is limiting when multiple variants or isoforms of a particular gene may exist naturally. It further eliminates endogenous expression control and disrupts other regulatory features of the native target locus. We therefore aimed to develop a universal co-selection method that: (1) does not require an exogenous selection marker, drug, or specialized equipment to enhance editing outcomes; (2) removes imprecisely edited cells in a cell-intrinsic manner; (3) introduces only synonymous changes at the selection locus, leaving the protein sequence unaltered; and (4) preserves natural regulatory control of the target locus.

To develop such a method, we turned to essential genes. Essential genes are required for cellular proliferation under normal environmental conditions and are considered intolerant to loss-of-function mutations. Among human cells, essential genes are indispensable for various cell-based processes, including cell cycle progression, nuclear trafficking, and metabolic processing [36]. A comprehensive list of pan-essential genes has been identified in both cancer cell lines and stem cells [37,38,39], which served as a basis for identifying candidate essential selection sites in our work.

Our co-selection strategy, hereafter referred to as HDRescue, uses essential genes as both positive and negative selection loci via HDR and NHEJ, respectively. Consequently, cell intrinsic selection for the primary, essential gene modification enriches the accompanying and desired passenger modification(s). Specifically, our approach installs synonymous blocking mutations within the protospacer and/or protospacer-adjacent motif of the essential genes *RAN* and *SF3B1* using CRISPR-Cas9 and an HDRescue donor. Negative selection occurs when NHEJ-mediated out-of-frame indel mutations at the essential site KO the gene, leading to cell death. However, cells that repair the DSB via HDR using the rescue donor undergo positive selection, leading to a substantial increase in editing at the second, desired target site.

Using HDRescue, we observed bulk KI efficiencies of up to 46% in induced pluripotent stem cells (iPSCs) and a substantial increase in the number of homozygously edited clonal lines. We further found that we can achieve a meaningful increase in KI mutations up to 800 bp. Additionally, we observed a minimal number of unwanted edits. Collectively, this work demonstrates that HDRescue is a robust, universal co-targeting strategy that will be widely applicable across cell lines to improve on-target editing while preserving the natural elements of the target locus.

## 2. Methods

### 2.1. Cell Culture and Reagents

BJFF.6 iPSCs (Cellosaurus, CVCL-VU02 [40]) and AN1.1 iPSCs (Washington University, St. Louis, MO, USA; T-019532) were cultured in StemFlex media (Gibco, Carlsbad, CA, USA), supplemented with 1% (*v*/*v*) antibiotic/antimycotic (Gibco) and 5% (*v*/*v*) CO_2_ in a humidified incubator at 37 °C. Edited iPSC pools were generated using CRISPR-Cas9 technology and homologous DNA donors. One million iPSCs were nucleofected (4D-Nucleofector™ X-unit; Lonza, Basel, Switzerland) per the manufacturer’s recommended protocol with pre-complexed ribonucleoprotein (RNPs) consisting of 100 pmol chemically modified single guide RNA (sgRNA; Integrated DNA Technologies, Coralville, IA, USA) (Supplementary Table S1) and 33 pmol 3XNLS *Sp*Cas9 protein (St. Jude Children’s Research Hospital [SJCRH] Protein Production Facility, Memphis, TN, USA) using P3 nucleofector solution and CA-137 program in 20-µL or 100-µL cuvettes. Control cell pools contained a non-targeting sgRNA and a non-targeting DNA donor to control for RNP, and donor concentrations added to an HDRescue editing experiment. Unless otherwise noted, editing rates were assessed on day 10 post-transfection.

U2OS cells (ATCC, HTB-96) were cultured in Dulbecco’s Modified Eagle Medium (DMEM) (Corning, 10-013-CV, Corning, NY, USA) supplemented with 10% (*v*/*v*) FBEssence, 1% (*v*/*v*) penicillin-streptomycin, and 1X GlutaMAX at 37 °C, 5% *v*/*v* CO_2_ in a humidified incubator. U2OS cell pools were generated by nucleofecting 500,000 cells as described above using P3 nucleofector solution (Lonza) and the CM-104 program in 20-µL cuvettes. Unless otherwise noted, editing rates were assessed on day 28 post-transfection.

HeLa cells (ATCC, CRM-CCL-2) were cultured in DMEM supplemented with 10% (*v*/*v*) FBEssence, 1% (*v*/*v*) penicillin-streptomycin, and 1X GlutaMAX at 37 °C, 5% *v*/*v* CO_2_ in a humidified incubator. HeLa cells pools were generated by nucleofecting 500,000 HeLa cells as described above using SE nucleofector solution (Lonza) and DS-150 program in 20-µL cuvettes. Unless otherwise noted, editing rates were assessed on day 16 post-transfection.

K-562 cells (ATCC, CCL-243) were cultured in Iscove’s Modified Dulbecco’s Medium (Gibco, 12440-053) supplemented with 10% (*v*/*v*) fetal bovine serum, 1% (*v*/*v*) penicillin-streptomycin, and 1X GlutaMAX at 37 °C, 5% *v*/*v* CO_2_ in a humidified incubator. K-562 cells (ATCC CCL-243) pools were generated by nucleofecting one million K562 cells as described above using P3 nucleofector solution (Lonza) and FF-120 program in 20-µL cuvettes. Unless otherwise noted, editing rates were assessed on day 10 post-transfection.

### 2.2. sgRNAs and DNA Donors

Alt-R sgRNAs and single-stranded oligodeoxynucleotide (ssODN) donors were synthesized by Integrated DNA Technologies (Coralville, IA, USA). Plasmid constructs were generated by GenScript (Piscataway, NJ, USA). Sequences of sgRNAs and DNA templates can be found in Supplementary Tables S1 and S2, respectively.

### 2.3. Cell Sorting and Flow Cytometry

One million U2OS cells, AN1.1 iPSCs, or BJFF.6 iPSCs were centrifuged at 100× *g* for 4 min and resuspended in phosphate-buffered saline -Ca++, -Mg++, + 2.5 mM EDTA. Flow cytometry data were acquired on a Cytek Aurora (Cytek, Fremont, CA, USA), equipped with SpectroFlo (SJCRH Flow Cytometry and Cell Sorting Shared Resource), and subsequently analyzed using FlowJo v11.1.1 (BD Life Sciences, Franklin Lakes, NJ, USA).

### 2.4. Genomic DNA Isolation and DNA Sequencing

One hundred thousand cells were centrifuged at 800× *g* for 5 min, resuspended in 100 μL genomic DNA quick extraction buffer (10 mM Tris, 2 mM EDTA, 0.2% Triton X-100, Proteinase K), and then processed at 65 °C for 15 min and 95 °C for 5 min. First-round PCR reactions were performed in a 10-μL reaction volume containing 0.25 μM forward and reverse primers with deep sequencing tags, 1 μL extracted genomic DNA, and 9 μL QuantaBio RepliQa HiFi ToughMix. The forward deep sequencing tag is “CTACACGACGCTCTTCCGATCT”. The reverse deep sequencing tag is “CAGACGTGTGCTCTTCCGATCT”. PCR conditions were as follows: 98 °C for 10 s, followed by 25 cycles of 98 °C for 10 s and 68 °C for 10 s. Round 2 PCR was performed as previously described [41]. PCR reactions were pooled to make a sequencing library and spiked with PhiX DNA before running the sample on a MiSeq i100 (Illumina, San Diego, CA, USA) to generate paired 2× 250-bp reads, as per the manufacturer’s instructions. Primer sequences without deep sequencing tags for each amplicon are listed in Supplementary Table S3. Samples were de-multiplexed using Illumina DNA/RNA Unique Dual Indexes Set A (Illumina). FASTQ files were generated and analyzed using CRIS.py [41].

### 2.5. Statistical Analysis

Statistics were performed with GraphPad PRISM version 11 software (Boston, MA, USA).

## 3. Results

### 3.1. HDRescue: Essential Gene Co-Editing Enriched HDR at the Target Locus

**Figure 1:**
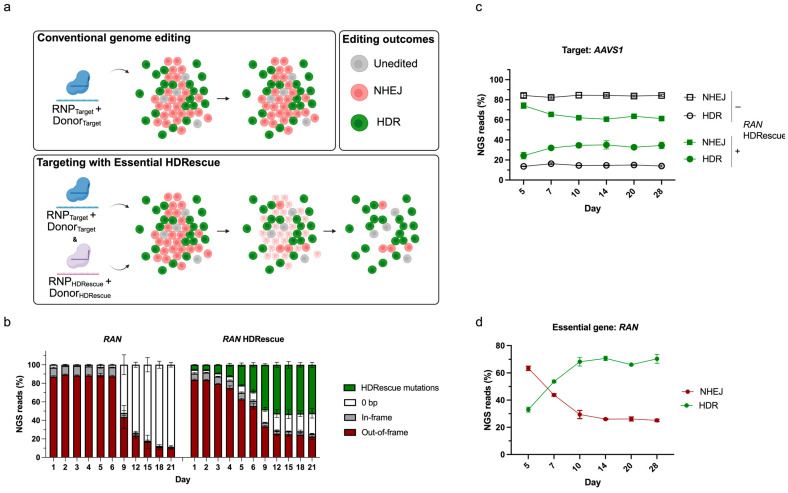
Essential HDRescue: Essential gene co-editing enriches homology-directed repair (HDR) at the target locus. (**a**) Schematic diagram of the HDRescue co-selection strategy in comparison to conventional genome editing. HDRescue employs two CRISPR–Cas9 ribonucleoprotein (RNP) complexes to generate double-stranded DNA breaks, one at the target site and one at an essential locus, along with two matched DNA donors to enable HDR at both locations. One donor installs a user-defined genome modification at the target site, while the second donor, the HDRescue template, introduces synonymous substitutions at an essential gene to prevent Cas9 from re-cutting that locus and preserve the wild-type protein sequence. (**b**) The *RAN* locus was tested using a cellular fitness assay to assess allelic ratios with and without the HDRescue single-stranded oligodeoxynucleotide donor in U2OS cells. The stacked bar plots show the percentage of in-frame indels, out-of-frame indels, 0-bp indels, and HDRescue mutations in the cell pool over time. (**c**) Non-homologous end-joining (NHEJ, squares) and HDR (circles) editing rates over time using conventional targeting (− HDRescue) and targeting with HDRescue (+ HDRescue) to install a 5-bp insertion at the target site, *AAVS1*, in U2OS cells. (**d**) NHEJ (red) and HDR (green) editing efficiencies at the *RAN* locus over time in U2OS cells co-targeted at *AAVS1*. Experiments were performed in triplicate; error bars represent the mean +/− s.d. The schematic was generated using Biorender.com. Abbreviation: NGS, next generation sequencing.

Our strategy to enhance HDR-mediated genome edits relies upon an essential gene as a co-selection locus. Unlike conventional HDR targeting, which typically uses one sgRNA-Cas RNP complex and a single DNA donor template, HDRescue requires two distinct RNP complexes and two separate DNA donors (Figure 1a).

One RNP and donor pair are directed to the desired target site while the second RNP and donor pair target an essential gene, which is used as a co-selection locus for HDR-mediated editing events. We predicted that cells edited by NHEJ that acquire out-of-frame mutations in the essential gene would stop proliferating and die. To enrich for HDR-edited cells, the HDRescue donor incorporates a synonymous mutation that preserves the wild-type (WT) protein sequence of the essential gene while rendering the target site resistant to Cas9 re-cleavage. This enables positive selection for cells that successfully completed HDR. The essential gene *RAN* was chosen as a candidate co-selection locus because it is required for cellular proliferation and has been shown to be essential across thousands of cell types [42,43]. We designed a highly efficient sgRNA targeting the *RAN* coding sequence to induce a genetic KO via the NHEJ pathway and used a Cellular Fitness (CelFi) assay to measure fitness and monitor indel formation over time [44]. The CelFi assay showed that introducing a targeted DSB within the coding sequence of *RAN* resulted in a high ratio of out-of-frame indels on day 1 in U2OS cells, as measured by next-generation sequencing (NGS) (Figure 1b). However, we observed a sharp decline in out-of-frame indels by day 9 post-transfection, and by day 18, 77% of these indels were depleted following *RAN* targeting. In contrast, an intronic *PPP1R12C* locus, hereafter referred to as the *AAVS1* safe harbor site, maintained a stable indel profile after day 3, when editing peaked (Supplementary Figure S1). An HDRescue single-stranded oligodeoxynucleotide (ssODN) donor template was designed to KI two synonymous mutations in the protospacer sequence of the *RAN* sgRNA (Supplementary Figure S2). When installed, this HDRescue KI conferred a selective advantage to *RAN*-targeted cells repaired by HDR. Over time, out-of-frame indels at the *RAN* locus were depleted, and more than 52% of all *RAN* alleles in the surviving cells carried the HDRescue synonymous mutations (Figure 1b).

As a proof-of-concept, we next asked whether HDRescue increases simultaneous, precise editing at the second targeted locus. To investigate this, we transfected U2OS cells with the *RAN* HDRescue reagents, as well as an RNP and DNA donor template designed to introduce a 5-bp sequence at a second, unrelated target locus, *AAVS1*. By day 14 post-transfection, we observed a 2.4-fold increase in the rate of 5-bp insertions at *AAVS1* with HDRescue compared to control cells, and this increase in HDR was maintained until the final day 28 timepoint (Figure 1c).

At the co-targeted *RAN* locus, we initially observed NHEJ mutations occurring more frequently than the HDRescue mutations; however, this pattern reversed halfway through the experiment (Figure 1d). On day 14, we observed the frequency of HDRescue mutations had reached ~70%, and this frequency was maintained through the final timepoint. Overall, these results support the use of essential genes as co-selection loci with HDRescue reagents, show that the synonymous edits are stable, and demonstrate that HDRescue can enrich HDR-mediated edits.

### 3.2. HDRescue Enabled Co-Selection of Desired Edits Across Multiple Cell Types Using Two Essential Gene Targets

**Figure 2:**
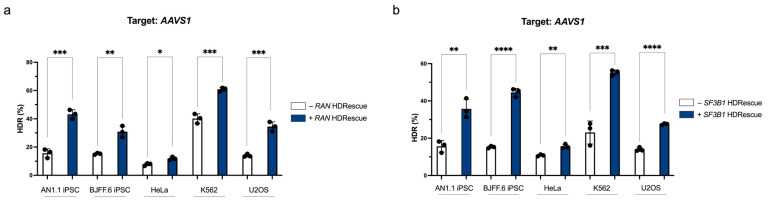
HDRescue enables co-selection of desired edits across multiple cell types using two essential gene targets. Knock-in of a 5-bp insertion at the *AAVS1* locus in BJFF.6 and AN1.1 induced pluripotent stem cells (iPSCs), as well as HeLa, K562, and U2OS cells with (+) or without (−) (**a**) *RAN* or (**b**) *SF3B1* HDRescue. Three biological replicates per experiment were performed; error bars represent mean +/− s.d. Significance was calculated using one-tailed Student’s *t*-tests, * *p* < 0.05, ** *p* < 0.01, *** *p* < 0.001, **** *p* < 0.0001.

To expand upon our proof-of-concept, we designed and validated a highly active sgRNA and cognate HDRescue ssODN donor targeting the essential splicing factor gene *SF3B1* for use in our co-targeting studies alongside *RAN*. We evaluated the efficiency of installing a 5-bp sequence at the *AAVS1* target site across five cell lines, including both cancer and iPSC lines, and compared conditions with and without HDRescue using *RAN* and *SF3B1* as the essential genes. Although HDR efficiency varied across cell lines, the pan-essential nature of *RAN* and *SF3B1* led us to predict that HDRescue would enhance targeting rates in all cell lines examined. In *RAN* HDRescue cells, we observed a 1.5- to 2.8-fold increase in HDR rates regardless of cell line compared to conventional genome targeting (Figure 2a).

Similarly, in *SF3B1* HDRescue cells, we observed a 1.4- to 2.9-fold increase in HDR rates regardless of cell line compared to single-targeted controls (Figure 2b). Notably, we achieved KI rates exceeding 20% in four of the five cell lines tested using HDRescue with both tested essential genes. In the absence of HDRescue, NHEJ frequencies at the *AAVS1* locus ranged from 55–86% (Supplementary Information Figure S3). HDRescue treatment resulted in reduced NHEJ rates, consistent with increased utilization of HDR and high cutting efficiency at this site. These results show that HDRescue improves HDR targeting across multiple cell lines and types, regardless of whether *RAN* or *SF3B1* HDRescue is used.

### 3.3. HDRescue Improved Multi-Allelic HDR Editing in Cell Clones

**Figure 3:**
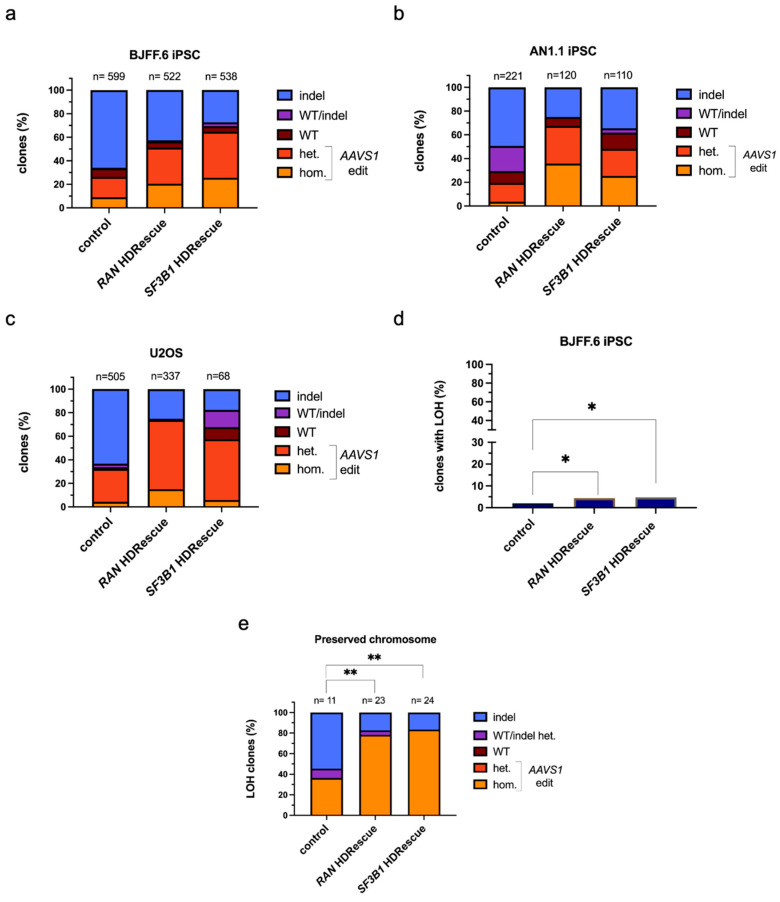
HDRescue improves multi-allelic homology-directed repair editing in cell clones. Allelic distribution of clones at the target site, *AAVS1*, with and without *RAN* or *SF3B1* HDRescue in (**a**) BJFF.6 induced pluripotent stem cells (iPSCs), (**b**) AN1.1 iPSCs, and (**c**) U2OS cells. (**d**) Percentage of BJFF.6 iPSC clones that experienced loss-of-heterozygosity (LOH). (**e**) Genotype of BJFF.6 clones on preserved chromosome. Data plotted is a percentage of total viable clones (n) from one biological replicate per condition. Abbreviations: WT, wild-type reads only; het., heterozygous (≥1 homology-directed repair [HDR] allele); hom., homozygous (all HDR alleles); indel, indel reads only; WT/indel, mixture of wild-type and indel reads. Significance was calculated using chi-square tests, * *p* < 0.05, ** *p* < 0.01.

Given successful fold-enrichment of HDR edits observed in bulk cell pools, we generated single-cell-derived clones from BJFF.6 and AN1.1 iPSCs and from U2OS osteosarcoma cells and then assessed the percentage of alleles edited at both the target locus (*AAVS1*) and the essential gene (*RAN* or *SF3B1*). Compared with a single-targeted control, both *RAN* and *SF3B1* HDRescue clones showed a higher proportion of cells with HDR-edited alleles and a lower proportion of cells with 100% indels at the *AAVS1* target site (Figure 3a–c, Supplementary Table S4).

Additionally, we observed a substantial increase in the number of clones containing homozygous HDR edits installed at the target site (2.0- to 9.9-fold) compared to non-HDRescue cells, regardless of cell type. U2OS cells showed the smallest increase in homozygous HDR events, likely due to their polyploid nature. We found very few clones with 100% indels at the essential gene, and these occurred only within a small subset of *SF3B1* HDRescue clones. This finding suggests that cells carrying NHEJ-mediated disruptions were selectively lost as a consequence of essential gene dependency (Supplementary Figure S4). Among the HDRescue clones that had 100% indels at *SF3B1*, each retained at least one in-frame allele, demonstrating that in-frame indels were tolerated at this locus (Supplementary Figure S5). Notably, there were many permutations of editing outcomes across multiple alleles, even in diploid cells (Supplementary Figure S6). We characterized desirable editing outcomes at essential *RAN* alleles as cells containing only HDR-mediated or non-edited alleles. However, cells with a combination of indel and WT or HDR alleles may be permissive for cellular proliferation. Out-of-frame indels on any allele at the *RAN* locus were always lethal and provided a means of predictable negative selection for cells edited imprecisely via NHEJ (Supplementary Figure S4). These results suggest that HDRescue not only enhances total HDR editing rates but also increases the proportion of homozygous-targeted HDR edits across the three cell lines tested.

### 3.4. Detection and Removal of Aberrant Clones Improve HDRescue Accuracy

Targeted DSBs can lead to larger chromosomal aberrations that might be missed by targeted amplicon sequencing approaches [45,46]. To investigate this further in our HDRescue clones and to reduce the risk of misclassifying clones as homozygous due to large deletions or complex rearrangements, we developed a PCR-based assay followed by NGS to detect a downstream heterozygous single-nucleotide polymorphism (SNP) located near, but outside, of the *AAVS1* target site in BJFF.6 clones (Supplementary Figure S7a). We found a low percentage (<5%) of clones classified as homozygous by NGS experienced loss of heterozygosity (LOH) at the tested SNP in all conditions (Figure 3d). Though the frequency was low, there was a significant increase in LOH between the control and HDRescue conditions, suggesting that HDRescue may select for cells with LOH. Furthermore, the small subset of clones that experienced LOH also exhibited a significant difference between HDR- or NHEJ-mediated outcomes on the preserved *AAVS1* allele (Figure 3e). This phenomenon may stem from the increased number of multiallelic HDR events observed in HDRescue clones. HDR is a slower repair mechanism than NHEJ and selecting cells with a higher propensity for HDR may increase the opportunity for LOH, resulting in the observed difference.

Another risk of genome editing is off-target cleavage [47]. Because HDRescue uses two RNPs, there is increased potential for off-target editing and translocations. Both the *SF3B1* and *RAN* sgRNAs target unique sites in the human genome, with no predicted off-target sites containing fewer than two mismatches. In addition, each tested essential gene sgRNA has only a single predicted off-target site with only two mismatches based on sequence homology (Supplementary Table S5). To assess potential off-target cleavage, we screened each clone shown in Figure 3 for editing at the single predicted off-target site containing only two mismatches to the target sequence for each essential-gene sgRNA. We detected no off-target cutting in AN1.1 clones edited with *SF3B1* sgRNAs, and less than 3% of all BJFF.6 and U2OS clones showed indels at the *SF3B1* predicted off-target site, *BDNF-AS* (Supplementary Figure S8, Supplementary Table S4). In contrast, a higher proportion of clones edited with the *RAN* sgRNA showed indels at the predicted off-target site, the *RAN* pseudogene *RANP6*, across all cell lines tested (Supplementary Figure S8, Supplementary Table S4). Despite this, we did not observe any change in indel ratios at the *RANP6* locus over time when using the CelFi assay, suggesting that editing at this off-target locus did not affect cellular proliferation (Supplementary Figure S8d).

Another major concern for co-targeting methods is the risk of translocations when two tandem DSBs occur in the same cell. To assess translocations after HDRescue, we developed PCR-based assays to detect translocations in both forward and reciprocal orientations. In theory, cells should not survive if a translocation disrupts an essential gene and no WT copies remain, particularly when using sgRNAs that cut early in the coding sequence of the essential gene. Not surprisingly, we detected limited evidence of forward or reciprocal translocation products in either the *RAN* or *SF3B1* HDRescue pools (Supplementary Figure S9). When we screened individual clones for translocations between the target site and the corresponding essential gene, we found no positive clones out of more than 1600 HDRescue clones screened (Supplementary Table S4). Taken together, these data suggest that the off-target edits and larger chromosomal abnormalities observed in some HDRescue clones can be detected effectively and the affected clones excluded, enabling reliable identification of high-quality, correctly targeted clones.

### 3.5. HDRescue Improved HDR Editing Rates Across Multiple Loci and Different Donor Formats

**Figure 4:**
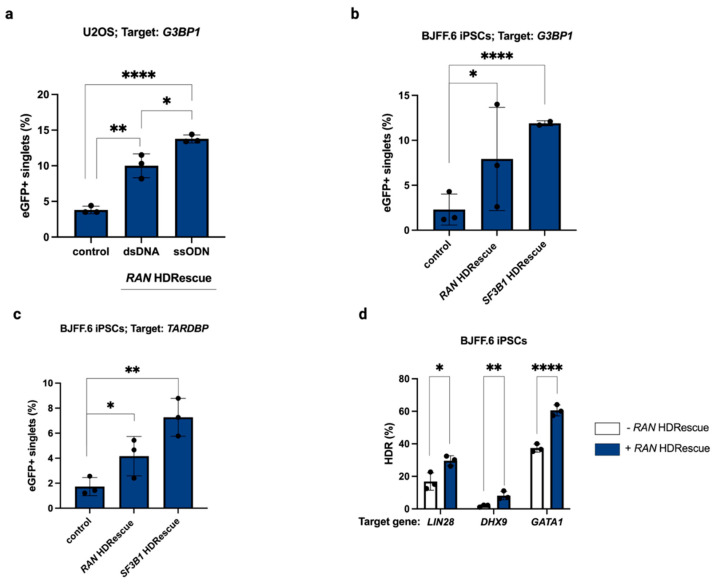
HDRescue improves knock-in editing rates across multiple loci and different donor formats. (**a**) Frequency of homology-directed repair (HDR) at target site *G3BP1* for a C-terminus enhanced GFP (eGFP) tag as measured by flow cytometry with two different *RAN* HDRescue DNA templates, double-stranded DNA (dsDNA), or single-stranded oligodeoxynucleotide (ssODN), in U2OS cells. (**b**) HDR frequency at target site *G3BP1* for a C-terminus eGFP tag as measured by flow cytometry in BJFF.6 induced pluripotent stem cells (iPSCs) with or without *RAN* or *SF3B1* HDRescue. (**c**) HDR frequency at target site *TARDBP* for a C-terminus eGFP tag as measured by flow cytometry in BJFF.6 iPSCs with *RAN* or *SF3B1* HDRescue. (**d**) HDR efficiency of three different targets using BJFF.6 iPSCs with or without *RAN* HDRescue. *n* = 3 biological replicates; error bars represent mean +/− s.d. Significance was calculated using one-tailed Student’s *t*-tests; * *p* < 0.05, ** *p* < 0.01, **** *p* < 0.0001.

Having demonstrated that we could generate HDR-modified cell pools and clones at higher yield using ssODNs at both the target site and essential gene, we next asked whether we could improve the yield of cells containing larger KI sequences, such as fluorescent protein tags, using double-stranded DNA (dsDNA) as the donor for the target gene. To test this, we first transfected U2OS cells with RNPs and the dsDNA donor to install a C-terminal enhanced green fluorescent protein (eGFP) tag at the target gene, *G3BP1*, with or without *RAN* HDRescue. By design, eGFP expression only occurs in correctly edited cells. In the absence of target-specific RNPs, no eGFP-positive cells were observed. For HDRescue, we compared two donor formats at the essential site: either an ssODN or a dsDNA plasmid. Using flow cytometry, we observed a 2.6-fold increase in the rate of eGFP-positive cells when dsDNA was used for the HDRescue donor and a 3.6-fold increase in eGFP-positive cells when an ssODN was used, compared to single-targeted control U2OS cells (Figure 4a).

Given the higher HDR rate when an ssODN donor was used as the HDRescue donor, we continued to use ssODNs for the essential gene in further studies. We transfected the *G3BP1*-eGFP tagging reagents with and without *RAN* or *SF3B1* HDRescue into BJFF.6 iPSCs and found a significant 3.5-fold and 10.8-fold increase in eGFP-positive *RAN* and *SF3B1* HDRescue cells, respectively, compared to single-targeted control cells (Figure 4b). Similarly, we found a significant 2.8- and 4.0-fold increase in the rate of eGFP tagging at the C-terminus of *TARDBP* when co-targeted with *RAN* or *SF3B1* HDRescue, respectively (Figure 4c).

Finally, we assessed HDR rates across different loci using HDRescue. We knocked in SNPs in genes *GATA1* and *DHX9* and a 5-bp insertion in gene *LIN28B,* with and without *RAN* HDRescue, and observed significant increases in HDR across all three genes tested (Figure 4d). By measuring the rates of HDR across different cell lines and different target genes, we have highlighted the utility of HDRescue across donor modalities (ssODN or dsDNA) and demonstrate its loci-agnostic capability to improve HDR rates overall.

## 4. Discussion

The efficiency of HDR following a site-directed DSB varies markedly with the edit, target loci, and cell type, and is generally outcompeted by NHEJ [48]. Here, we demonstrated HDRescue, a novel CRISPR-based co-selection strategy that pairs NHEJ-mediated disruption and selective elimination of NHEJ-edited cells with HDR-mediated installation of rescuing synonymous SNPs at an essential locus. HDRescue enables strong enrichment of user-defined HDR edits at an unrelated target site, allowing efficient recovery of precisely engineered cells in a cell-intrinsic, marker-free manner. These results support previous findings that cells capable of completing one genomic manipulation are more likely to complete a second, independent manipulation when both rely on similar DNA repair mechanisms [21,29,30,31,32,34]. This co-selection is likely reliant on the cell cycle phase at the time of genome editing [48]. Cycling cells in late S or early G2 phases have a higher HDR frequency than those in other phases, and this has been used as a basis for increasing HDR frequency via cell cycle control [49,50]. However, our approach circumvents the need to chemically arrest cells to improve HDR rates.

HDRescue not only increases overall HDR editing in bulk cell populations but also increases the frequency of homozygous HDR mutations in clonal populations, enabling higher-throughput generation of engineered homozygous clones, which is particularly valuable in polyploid cell lines. This high rate of homozygous KI clones suggests that biallelic editing is not random but is something potentially selected for. HDRescue is a cost-, time-, and labor-saving method primarily suited for generating isogenic cell clones with precise HDR-engineered mutations, but it can also be used to create enriched pools of edited cells when downstream applications tolerate mixed editing outcomes.

Compared with other co-selection methods for HDR editing, HDRescue offers several key advantages. The use of pan-essential genes, such as *RAN* and *SF3B1*, as co-selection loci enables a universal co-selection method, as the loss of pan-essential genes from out-of-frame indels repaired by NHEJ results in cell death. Our technique avoids cell cycle control and pharmacological inhibition of NHEJ to improve HDR rates, enabling the generation of HDR-enriched cell populations without the risk of triggering genomic instability or replication stress from the addition of cell cycle-modulating drugs [51,52]. HDRescue can enrich for small inserts and larger fluorescent tags without exogenous or episomal selection markers, specialized techniques, or equipment. Furthermore, this strategy does not require a drug or toxin to induce selection and does not introduce bacterial elements into the selection locus, enabling the generation of relatively naïve cells for preclinical research and in vitro modeling. Importantly, HDRescue allows the target gene to remain under its own regulatory elements. Compared with alternative genome editing strategies, such as prime or base editing, selection using HDRescue does not require optimization of pegRNA design and poses fewer risks of bystander edits. This technique stands out for its simplicity, as only one or two nucleotide changes are required to introduce synonymous mutations at the essential gene selection locus. We did not detect any clones with translocation products between the target and essential sites out of hundreds of screened clones. We predict that this translocation rate is lower than that for translocations between two non-essential loci, since cells lacking expression of an essential gene are not expected to proliferate.

Regardless of its advantages, each co-selection method comes with its own limitations. Despite cell-intrinsic positive and negative selection, WT and permissive-indel clones can remain because cells with at least one in-frame or WT allele can retain viability. For this reason, essential gene sgRNAs should be vetted for optimal, near-100% cutting efficiency with strong out-of-frame indel profiles to provide the most selective negative pressure. Furthermore, we tested two essential gene targets to provide options to enhance HDR across a range of targets and cell lines. While we observed HDR enrichment with both essential-gene targets, further empirical optimization may be necessary depending on the editing context, as factors such as DSB formation kinetics, NHEJ and HDR dynamics, cell-doubling time, essential gene expression, and protein half-life vary across cell types. Dependence on essential genes as selection loci may vary across different cell types or physiological conditions, as essential genes are not a simple, binary classification. Therefore, we recommend vetting essential gene-specific dependencies before using HDRescue, particularly for clinically relevant cell types that may harbor unique SNPs at essential loci or whose essential gene product may have different isoforms or post-translational modifications. While this work focused on the essential genes *RAN* and *SF3B1*, there are many other widely conserved essential genes that could be utilized, and selection could also be tailored to genes essential in a specific cell line. Since two DSBs are introduced simultaneously, we acknowledge the possibility of increased genomic instability, particularly for sgRNAs that have high off-target activity. While this work did not include a non-biased in vitro method to detect off-target cutting, we limited our sgRNA design to those that had at least two mismatches between the gRNA target site and any other site in the genome. We found high off-target cutting at the *RAN* pseudogene *RANP6* due to the high homology between the two sequences, which could be mitigated by optimizing sgRNA design or by using high-fidelity Cas9 variants. Furthermore, we recommend that the essential gene should never be on the same chromosome as the targeted locus, as this could lead to a large deletion between the two concurrent DSBs. Finally, HDRescue enriches for HDR-competent cells. Although this phenomenon is likely dependent on the cell cycle phase at the time of editing, we do not discount the possibility of selection bias towards HDR-competent cells, which could, in turn, impact downstream functional studies using HDRescue cells.

This simple method can be broadly applied across cell lines and targeted sites to increase the frequency of genome-editing events in mammalian cells by harnessing cellular dependencies. HDRescue is easy to implement, as it only requires an additional RNP and a cognate ssODN to induce selection at the essential gene. Although we limited our studies to CRISPR-Cas9, it should be compatible with any nuclease that generates a site-directed DSB. Future studies could include the installation of common, naturally occurring SNPs as HDRescue synonymous mutations within the coding sequences of essential genes, thereby further reducing the risk of unintended biological consequences from generating HDRescue-modified cells. Furthermore, we predict that the stringency of selection could be further optimized by titrating the ratio of the target donor to the HDRescue donor, as we hypothesize that a lower amount of the HDRescue donor would provide greater selective pressure.

Collectively, our findings establish HDRescue as a powerful, cell-intrinsic co-selection approach that enhances HDR-mediated genome editing across multiple loci, cell types, and donor formats. HDRescue consistently improves HDR frequencies over 2-fold in both bulk pools and clonal populations, increases the recovery of homozygous edits, and maintains a low, manageable burden of off-target activity and structural variation. Importantly, this approach reduces the number of clones needing to be screened to identify correctly edited cells, providing significant savings in time, labor, and cost. This simple and robust technique delivers high-efficiency, precise genome editing, and we expect that HDRescue will help accelerate discoveries across a wide range of biological and biomedical research.

## Data Availability

All sequencing data generated during this study are provided as fastq files. The SRA data generated in this study have been submitted to the NCBI BioProject database (https://www.ncbi.nlm.nih.gov/bioproject/ (accessed on 10 March 2026)) under accession number PRJNA1450718. Relevant materials are available upon request to shondra.miller@stjude.org.

## Supplementary Materials

The following supporting information can be downloaded at: https://www.mdpi.com/article/10.3390/cells15090768/s1, Figure S1: Cellular fitness assay of the AAVS1 locus; Figure S2: Schematic of targeted RAN locus; Figure S3: Rates of NHEJ at the AAVS1 locus with and without HDRescue; Figure S4: HDRescue clonal editing outcomes; Figure S5: Assessment of indel profile in SF3B1 HDRescue clones with 100% indels at SF3B1; Figure S6: Schematic of diploid editing outcomes permutations; Figure S7: Assessment of loss-of-heterozygosity in BJFF.6 HDRescue clones; Figure S8: Assessment of off-target cutting in clones; Figure S9: Assessment of translocations between target and essential sites via PCR and gel electrophoresis for AN1.1 iPSCs; Table S1: Guide sequences; Table S2: Homology-directed repair donor sequences; Table S3: Primer sequences for Illumina sequencing; Table S4: Editing outcomes in HDRescue clones; Table S5: In silico–predicted off-target sites for sgRNAs with ≤2 mismatches.
