# Supplementary material for: Essential HDRescue: A Co-Targeting Strategy to Enhance Precision Genome Editing by Co-Editing Essential Genes
Source: Cells. 2026 Apr 24;15(9):768. doi: 10.3390/cells15090768 (PMC13162901; doi:10.3390/cells15090768)
Supplement: Supplementary file 1 [file cells-15-00768-s001.zip › cells-4226774-supplementary.pdf]

Supplementary Information for

Essential HDRescue: A Co-Targeting Strategy to Enhance Precision Genome Editing by Co-Editing Essential Genes

This PDF file includes:

Figures S1 to S9

Tables S1 to S5

Corresponding author: shondra.miller@stjude.org

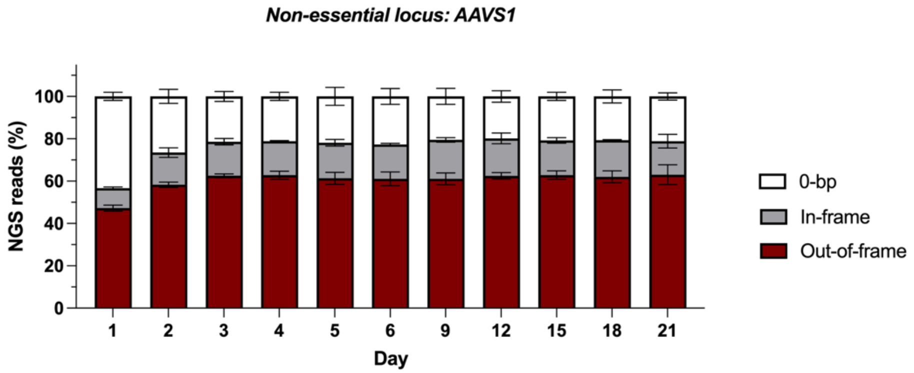

Figure S1 Cellular fitness assay of the *AAVS1* locus. The *AAVS1* locus, a non-essential intronic safe harbor site locus, was tested using a cellular fitness assay to assess cellular dependency in U2OS cells. The stacked bar plots show how the percentage of in-frame indels, out-of-frame indels, and 0-bp alleles in the cell pool change over time. Experiment was performed in triplicate; error bars represent the mean +/- s.d.

DNA (WT) 5’ AAC ATC CCC ATT GTG TTG TGT GGC 3’

DNA (HDRescue) 5’ AAC ATC CCC ATT GTG CTC TGT GGC 3’

Protein N’ Asn Ile Pro Ile Val Leu Cys Gly C'

Figure S2 Schematic of targeted *RAN* locus. Wild-type (WT) and HDRescue sequences shown with corresponding amino acid sequence. PAM sequence is highlighted in red. Protospacer sequence is underlined in black. Silent HDRescue mutations are highlighted in blue. Targeted amino acid (leucine) is highlighted in green.

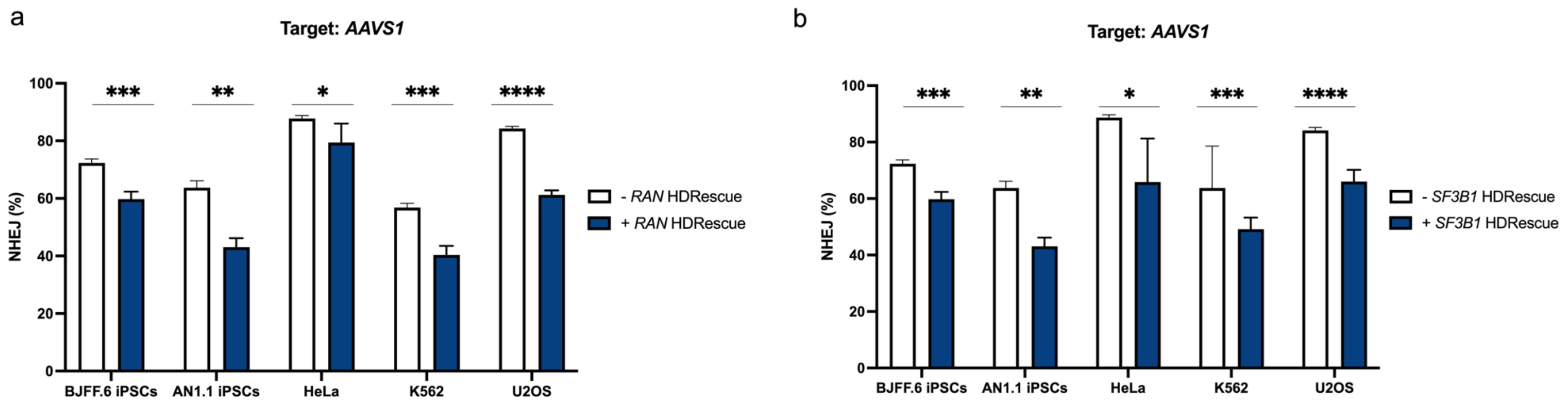

Figure S3 Rates of NHEJ at the *AAVS1* locus with and without HDRescue. Rates of non-homologous end joining at the *AAVS1* locus in BJFF.6, AN1.1, K562, and U2OS cells with (+) or without (-) (**a**) *RAN* or (**b**) *SF3B1* HDRescue. Three biological replicates per experiment were performed; error bars represent mean +/- s.d. Significance was determined using one-tailed Student's *t*-tests, $^{*}P<0.05$, $^{**}P<0.01$, $^{***}P<0.001$, $^{****}P<0.0001$.

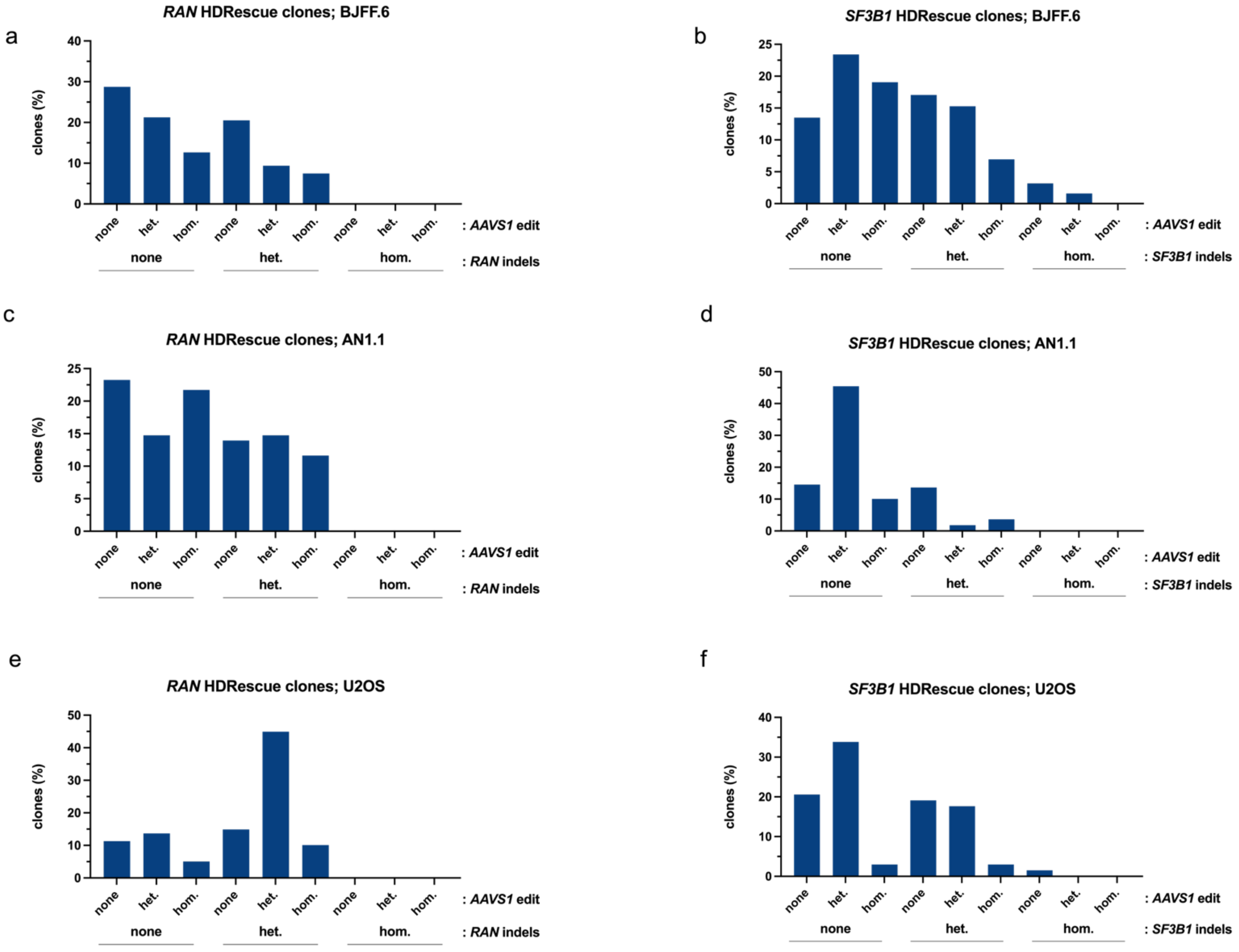

Figure S4 HDRescue clonal editing outcomes. HDRescue clones stratified by percentage of HDR edits at the target, *AAVS1,* vs. the percentage of indels at the essential gene for **(a)** *RAN* HDRescue in BJFF.6 iPSCs, **(b)** *SF3B1* HDRescue in BJFF.6 iPSCs, **(c)** *RAN* HDRescue in AN1.1 iPSCs, **(d)** *SF3B1* HDRescue in AN1.1 iPSCs, **(e)** *RAN* HDRescue in U2OS cells, and **(f)** *SF3B1* HDRescue in U2OS cells. Abbreviations: het. - heterozygous, hom. - homozygous

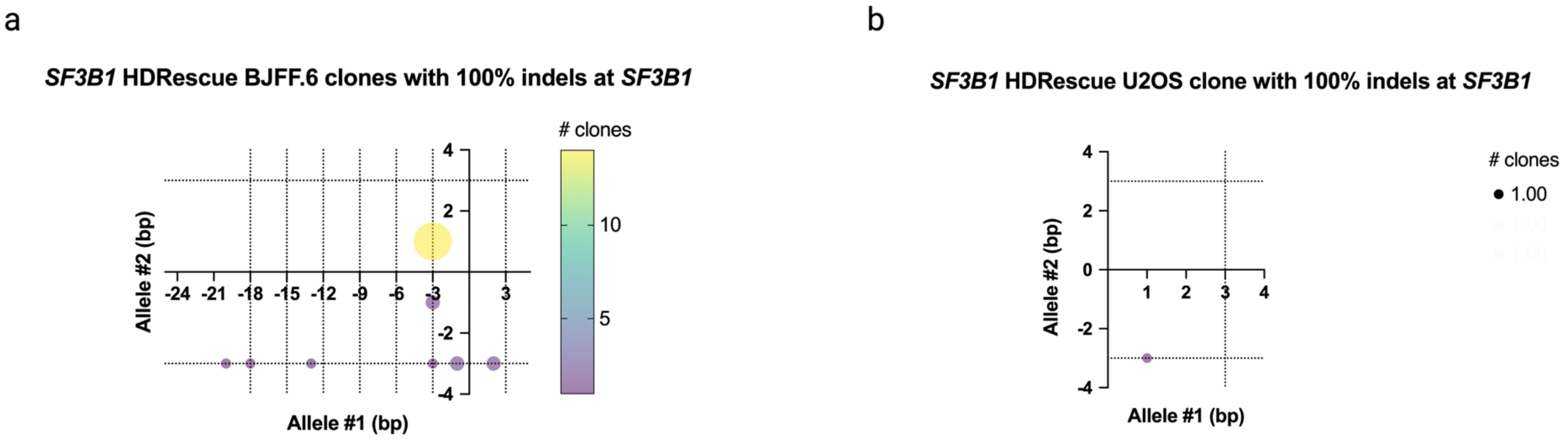

Figure S5: Assessment of indel profile in *SF3B1* HDRescue clones with 100% indels at *SF3B1* Bubble plot of indel distributions per allele in **(a)** BJFF.6 clones and a **(b)** U2OS clone. The color and size of bubbles indicate the number of clones per genotype. In-frame indels are marked with a dotted line.

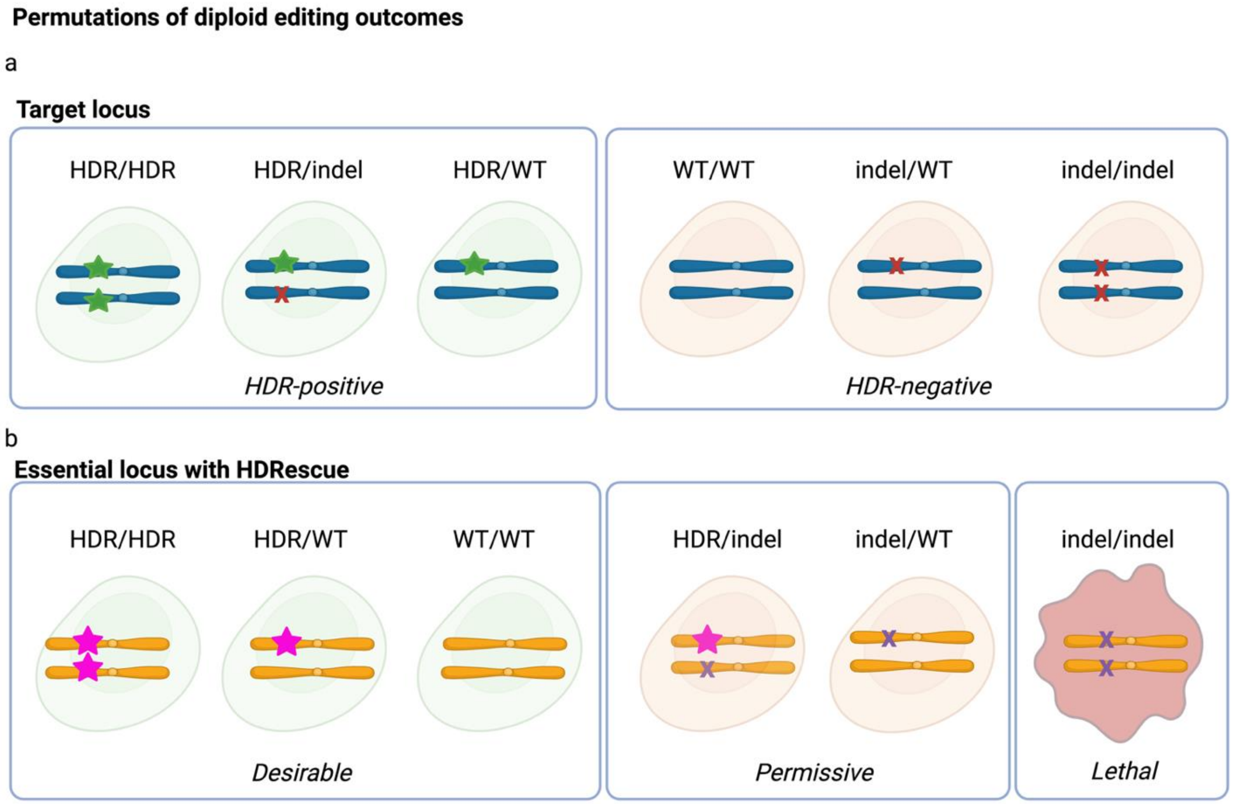

Figure S6 Schematic of diploid editing outcomes permutations at (**a**) the target locus and (**b**) the essential locus with HDRescue. Cells with desirable editing outcomes are shaded green and are comprised of primarily homology-directed repair (HDR) editing events whereas cells with undesirable editing outcomes are shaded orange and are comprised of primarily non-homologous end-joining (NHEJ) editing events. In (**a**), green stars denote precise HDR events at the target locus. A red "x" denotes an NHEJ event at the target locus. In (**b**), cells highlighted in red contain 100% indels at the essential gene. Pink stars denote precise HDRescue mutations at the essential locus. A purple "x" denotes an NHEJ event at the essential locus. Schematic made with Biorender.com

a

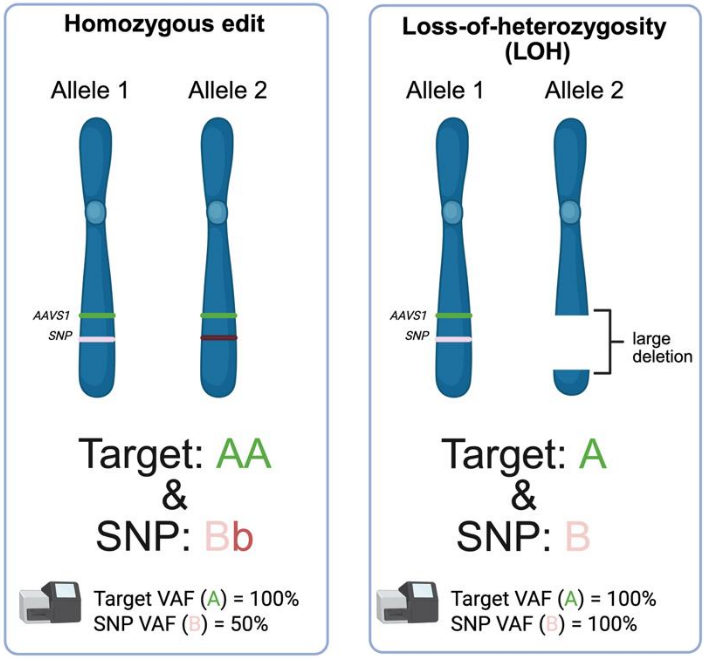

Figure S7: Assessment of loss-of-heterozygosity in BJFF.6 HDRescue clones. (a) Schematic of loss-of-heterozygosity (LOH) assay for either a true homozygous edit at the target site, *AAVS1*, (left) or LOH (right). The target *AAVS1* edit (AA) is illustrated as a green bar. Five kilobases downstream from the target sites is a heterozygous single-nucleotide polymorphism (SNP) denoted as Bb and illustrated with a dark red and pink bar. If LOH occurs from a large deletion, targeted amplicon sequencing shifts the variant allele frequency (VAF) from 50% per either SNP (B or b) to 100% per either SNP (B or b). Schematic made by Biorender.com.

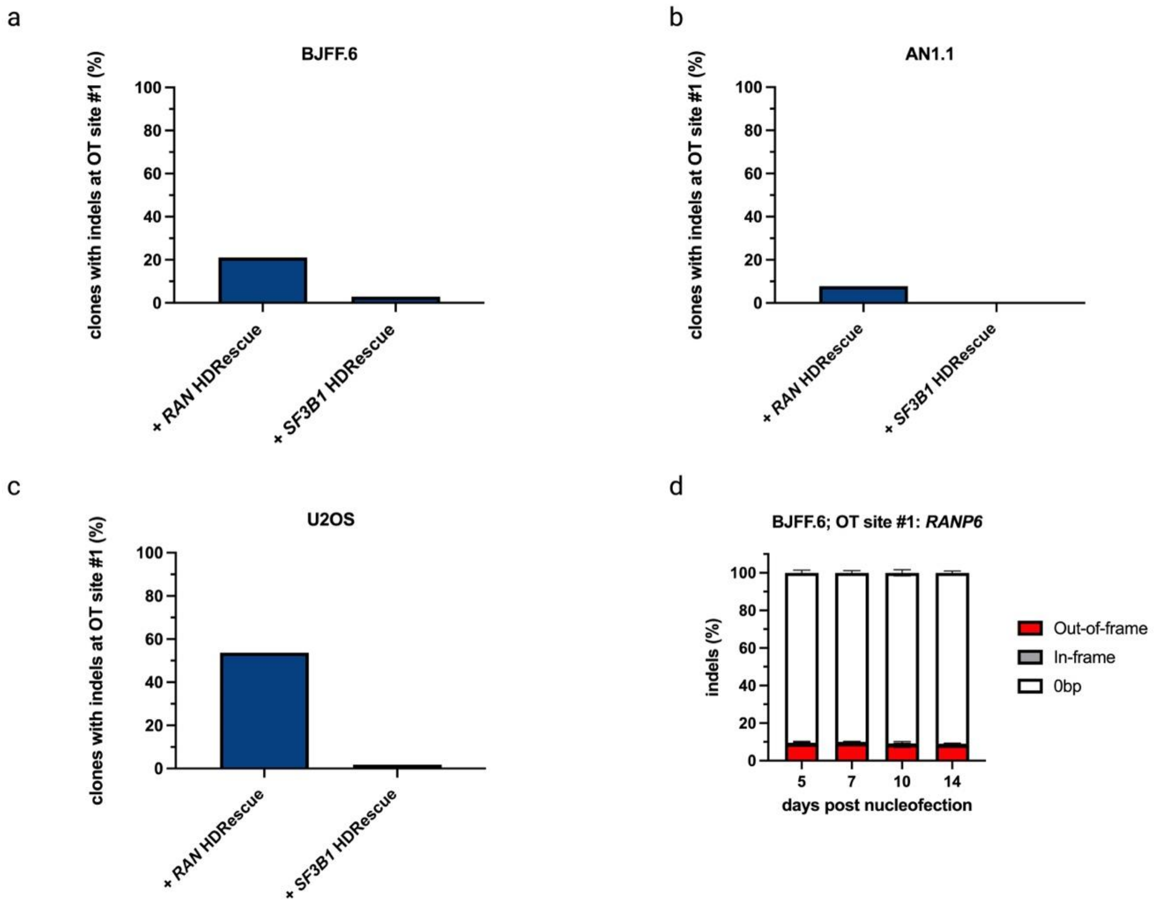

Figure S8 Assessment of off-target cutting in clones. Percentage of **(a)** BJFF.6, **(b)** AN1.1, and **(c)** U2OS clones that have indels at the top predicted off-target (OT) site for each essential gene sgRNA based on homology. **(d)** Cellular fitness assay at OT site #1 for *RAN* pseudogene, *RANP6,* illustrates changes in in-frame indels, out-of-frame indels, and 0-bp alleles over time.

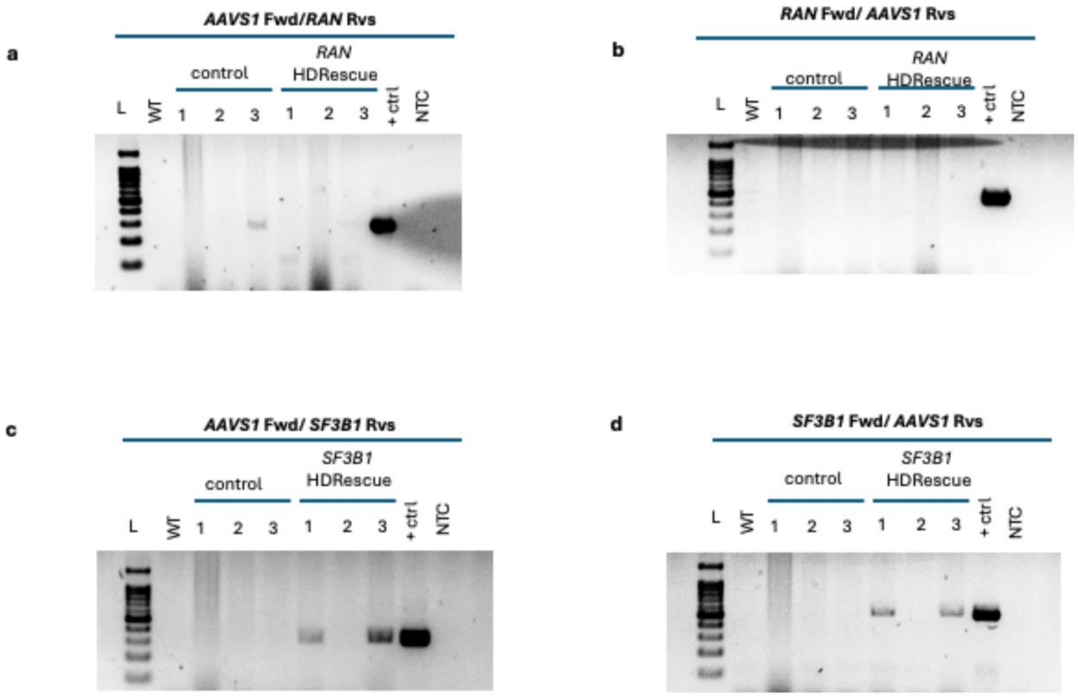

Figure S9 Assessment of translocations between target and essential sites via PCR and gel electrophoresis for AN1.1 iPSCs. Translocation PCRs between **(a)** *AAVS1* and *RAN* and **(b)** *RAN* and *AAVS1* for AN1.1 control and *RAN* HDRescue iPSC pools at day 5 post-transfection. Translocation PCRs between **(c)** *AAVS1* and *SF3B1* and **(d)** *SF3B1* and *AAVS1* for AN1.1 control and *SF3B1* HDRescue iPSC pools at day 5 post-transfection. Abbreviations: L = 100-bp ladder; WT= wild-type; + ctrl = positive control gBlock; NTC = no template control; Fwd = forward primer; Rvs = reverse primer; R HDRescue = *RAN* HDRescue; S HDRescue = *SF3B1* HDRescue.

Table S1: Guide sequences

Gene Name	NCBI Gene ID	gRNA ID	Spacer sequence (5' to 3')
PPP1R12C (AAVS1)	54776	g2	ACCCCACAGUGGGGCCACUA
RAN	5901	g3	AACAUCCCCAUUGUGUUGUG
SF3B1	23451	g6	UGGAUGAGCAGCAGAAAGUU
G3BP1	10146	g10	UGGGAAGGGGGCUUGCGCC
DHX9	1660	g5	GCGACAAAACCAAGUGGGUG
LIN28B	389421	g10	CAGUUGGAGCAUAAGUUAAG
GATA1	2623	g3	CCAUUGCUCAACUGUAUGGA
TARDBP	23435	g9	UGGCUGGGGAAUGUAGACAG

Table S2: Homology-directed repair donor sequences

Gene Name	gRNA ID	Donor Sequence (5' to 3')
PPP1R12C	g2	cctaaggatggggcttttctgtcaccaatcctgtccctagGATTCtggccccactgtggggtggaggggacagataaaagtaccc
RAN	g3	tagagatctggtacgagtgtgtgaaaacatccccattgtgCTTtgtggcaacaaagtggatattaaggacaggaaagtgaagg
SF3B1	g6	ttcgataccataaggagttgctgcttcagccaaggcagcaatggccaaagcactgatggtCcggaCtttctgctgctcatccacaagacctacaaaaccaaa cacaggttttaactatgccccaacatt
G3BP1	g10	gaaggtggcaattttttttattttttattttttattgggacggagtctcactctgtcgcccatgctggagtgcagtggcacgaccttggctcaccacaacttccgccacc cgggttcaagttgcactccagtctgggtgacgagtgaaactctgtctcattaaaaaaacaaacaaaaaactgaactgtgagaacttagctgatttataatctgct ggttctttttttttttctgagtcggagtctcgctctttttcccagactggagtacaatggcgtgatcttggctcactgcaacctccgcctcctgggttcaagcaattctcctg cctcagcctccttgaatagctggaactacaggcgtgtgtcaccatgtctggctaattttgtgtttttttagtagagacggggtttcaccatgttggtcaggctggtcttg aactcctgacctcgtaatccacccgtctcggcctcccaaagtgctgggattacaggcatgagtcaccgtgcccagcctctgctggttcattattacagctttctttat ctttgatagtgatagccagctcatcagtactcttaagtctggtcaccttgattcttacagcccatcatgttcagaggtgaggtccgtctgaatgtcgaagagaagaa gactcgagctgccagggaaggcgaccgacgagataatcgccttcggggacctggaggccctcgaggtgggctgggtggtggaatgagaggccctccccg tggaggcatggtgcagaaaccaggatttggagtgggaagggggcttgcgccaAgAcagggtggttctggtggtggttctggtggtATGGTGAGCAA GGGCGAGGAGCTGTTCACCGGGGTGGTGCCCATCCTGGTCGAGCTGGACGGCGACGTAAACGGCCACAA GTTCAGCGTGTCCGGCGAGGGCGAGGGCGATGCCACCTACGGCAAGCTGACCCTGAAGTTCATCTGCAC CACCGGCAAGCTGCCCGTGCCCTGGCCCACCCTCGTGACCACCCTGACCTACGGCGTGCAGTGCTTCAG CCGCTACCCCGACCACATGAAGCAGCACGACTTCTTCAAGTCCGCCATGCCCGAAGGCTACGTCCAGGAG CGCACCATCTTCTTCAAGGACGACGGCAACTACAAGACCCGCGCCGAGGTGAAGTTCGAGGGCGACACCC TGGTGAACCGCATCGAGCTGAAGGGCATCGACTTCAAGGAGGACGGCAACATCCTGGGGCACAAGCTGGA GTACAACTACAACAGCCACAACGTCTATATCATGGCCGACAAGCAGAAGAACGGCATCAAGGTGAACTTCA AGATCCGCCACAACATCGAGGACGGCAGCGTGCAGCTCGCCGACCACTACCAGCAGAACACCCCCATCG GCGACGGCCCCGTGCTGCTGCCCGACAACCACTACCTGAGCACCCAGTCCGCCCTGAGCAAAGACCCCA ACGAGAAGCGCGATCACATGGTCCTGCTGGAGTTCGTGACCGCCGCCGGGATCACTCTCGGCATGGACGA GCTGTACAAGtgaatcttcatggatcttcatgcagccatacaaaccctggttccaacagaatggtgaattttcgacagcctttggtatcttggagtatgaccc cagtctgttataaactgcttaagtttgtataattttactttttttgtgtgttaatggtgtgtgctccctctccctctcttccctttcctgacctttagtctttcacttccaattttgtgg aatgatattttaggaataacggacttttaaagaagcaaaaaaaaaagactgaatttccttgcttactttgcatatacagactggatttttttttttttttacagccatttccc caaaggaatgtcttgcatattactgacatttggtatgtttcattcattggaatatttcttattttctacgtgtttgaaaagcctgtaagaaatacaggatttgataatattttg aaggcaggaaaaacccaaattgtttcttctttgagagtcatgactaccttctggtgtggagaaattgccattggaaaatttgacaattttgattctcactggtatgttta aaaactgaataaaaggaatagaatttttttttgataaaggatcacaaaacaattctaaaacctaactgtttttaccattgaaatttaaattgtgataataggttttaaa

		tgtctagaatgcaactgataggcttttcttgaactgttagtttttttgaagtagttttttcatgtttaatttgtatttgtaaaaaaacaaaaagcaaaaaaattcccaaaac<br>ccagataacaaccagagcaaaactgttgt
DHX9	g5	ggattccagttggattgtggaggtgaccaaggaaccacaccGacttggttttgCCTctgAGCtggttcgaactgagccaatttgccaatgttgagtgcaact
LIN28B	g10	ttggagtaagttccatgttaatactcaccactgaggatatCGACGCaGcacttaacttatgctccaactgatgtaaaacactgcctaaatggc
GATA1	g3	cgtaggcccagccggcatatggtgagccccctgggatcccTtcAatacagttgagcaatgggtacacctgaaagactgttgggg
TARDBP	g9	cacagtcctcccaccttggcctcccaaagcgctaggattacaggcatgagccaccatgcccagcctatgtcttttgaaaatcgactgaaatatcactgctgctgt<br>taataaaactaaaagctgtattgggggtttaaatgaaatgagtgttcattgcttatttttcctctggctttagataaattaatgcttgtaatctaagttttgttgctactttaa<br>atatatgaatcagtggtttaatcttctttgtttacatcccttatttcttatagattgcgcagtctctttgtggagaggacttgatcattaaaggaatcagcgttcatatatcc<br>aatgccgaacctaagcacaatagcaatagacagttagaaagaagtggaagatttggtggtaatccaggtggctttgggaatcagggtggatttggtaatagc<br>agagggggtggagctggtttgggaaacaatcaaggtagtaatatgggtggtgggatgaactttggtgcgttcagcattaatccagccatgatggctgccgccc<br>aggcagcactacagagcagttggggtatgatgggcatgttagccagccagcagaaccagtcaggcccatcgggtaataaccaaaaccaaggcaacatg<br>cagagggagccaaaccaggccttcggttctggaaataactcttatagtggctctaattctggtgcagcaattggttggggatcagcatccaatgcagggtcggg<br>cagtggttttaatggaggctttggctcaagcatggattctaagtcttctggctggggaatgggtggttctggtggtggttctggtggtATGGTGAGCAAGG<br>GCGAGGAGCTGTTCACCGGGGTGGTGCCCATCCTGGTCGAGCTGGACGGCGACGTAAACGGCCACAAGT<br>TCAGCGTGTCCGGCGAGGGCGAGGGCGATGCCACCTACGGCAAGCTGACCCTGAAGTTCATCTGCACCA<br>CCGGCAAGCTGCCCGTGCCCTGGCCCACCCTCGTGACCACCCTGACCTACGGCGTGCAGTGCTTCAGCC<br>GCTACCCCGACCACATGAAGCAGCACGACTTCTTCAAGTCCGCCATGCCCGAAGGCTACGTCCAGGAGCG<br>CACCATCTTCTTCAAGGACGACGGCAACTACAAGACCCGCGCCGAGGTGAAGTTCGAGGGCGACACCCTG<br>GTGAACCGCATCGAGCTGAAGGGCATCGACTTCAAGGAGGACGGCAACATCCTGGGGCACAAGCTGGAGT<br>ACAACTACAACAGCCACAACGTCTATATCATGGCCGACAAGCAGAAGAACGGCATCAAGGTGAACTTCAAG<br>ATCCGCCACAACATCGAGGACGGCAGCGTGCAGCTCGCCGACCACTACCAGCAGAACACCCCCATCGGC<br>GACGGCCCCGTGCTGCTGCCCGACAACCACTACCTGAGCACCCAGTCCGCCCTGAGCAAAGACCCCAAC<br>GAGAAGCGCGATCACATGGTCCTGCTGGAGTTCGTGACCGCCGCCGGGATCACTCTCGGCATGGACGAG<br>CTGTACAAGtgaacagtggggttgtggttggttggtatagaatggtgggaattcaaatttttctaaactcatggtaagtatattgtaaaatacatatgtactaa<br>gaattttcaaaattggtttgttcagtgtggagtatattcagcagtatttttgacatttttctttagaaaaaggaagagctaaaggaattttataagttttgttacatgaaag<br>gttgaaatattgagtggttgaaagtgaactgctgtttgcctgattggtaaaccaacacactacaattgatatcaaaaggtttctcctgtaatattttatccctggacttg<br>tcaagtgaattctttgcatgttcaaaacggaaaccattgattagaactacattctttaccccttgttttaatttgaaccccaccatatggattttttccttaagaaaatct<br>ccttttaggagatcatggtgtcacagtgtttggttcttttgttttgtttttaacacttgtctcccctcatacacaaaagtacaatatgaagccttcatttaatctctgcagttc<br>atctcatttcaaatgtttatggaagaagcacttcattgaaagtagtgctgtaaatattctgccataggaatactgtctacatgctttctcattcaagaattcgtcatcac<br>gcatcacaggccgcgtctttgacggtgggtgtcccatttttatccgctactctttatttcatggagtcgtatcaacgctatgaacgcaaggctgtgatatggaaccag<br>aaggctgtctgaacttttgaaaccttgtgtgggattgatgg

*dsDNA donors are in pUC57 backbone, homology arms and insert shown

Table S3: Primer sequences for Illumina sequencing

Gene Name	gRNA ID	Forward Primer (5' to 3')	Reverse Primer (5' to 3')
PPP1R12C	g2	CCAACCCGGGCCCCTATGTCCACTT	CCTGCCAAGCTCTCCCTCCCAGGAT
RAN	g3	AGGTGACTCGTTGAGGTCTAAG	CGGTGGAAGACAATGGATTTCG
SF3B1	g6	TGGGGAGATAAATGGAAAGGCATAGCTC	CCTCTGTGTTGGCGGATACCCTTCCA
G3BP1	g10	ACTCGAGCTGCCAGGGAAGGCGACC	TCCAAGATACCAAAGGCTGTCGAAA
DHX9	g5	TCTTGAGATTTTGCCCCCGGT	TATTGCACTCACAAAAGCCAGAG
LIN28B	g10	TGTTTGCCTTTCTGTACACTCTGGGT	CACAAGCTTGCACAATCCCTGCTGG
GATA1	g3	GCCTGTGGAAAAGCTGGGAACTTGGC	GCTGGTGCTGCCTTTTCCATCCAGA
TARDBP	g9	AAACCAGGCCTTCGGTTCTGGAAAT	GAGTGGTTGAAAGTGAACTGCTGTTTGC

*Partial Illumina adaptor overhangs not shown but can be found in the methods section

Table S4: Editing outcomes in HDRescue clones

	BJFF.6			AN1.1			U2OS		
**Clones with:**	**% control (#)**	**% *RAN* HDRescue (#)**	**% *SF3B1* HDRescue (#)**	**% control (#)**	**% *RAN* HDRescue (#)**	**% *SF3B1* HDRescue (#)**	**% control (#)**	**% *RAN* HDRescue (#)**	**% *SF3B1* HDRescue (#)**
**Homozygous target edit**	9.0 (54)	20.5 (107)	25.5 (137)	3.6 (8)	35.8 (43)	25.5 (28)	4.4 (22)	15.1 (51)	5.9 (4)
**Heterozygous target edit**	17.2 (103)	30.7 (160)	39.2 (211)	15.8 (35)	31.7 (38)	22.7 (25)	27.9 (141)	58.8 (198)	51.5 (35)
**All indels at target**	66.0 (395)	42.7 (223)	27.5 (148)	49.3 (109)	25 (30)	34.6 (38)	63.4 (320)	25.2 (85)	17.7 (12)
**All WT at target**	7.4 (44)	5.2 (27)	4.7 (25)	10 (22)	7.5 (9)	13.6 (15)	1.4 (7)	0.9 (3)	10.3 (7)
**Indel/WT at target**	.5 (3)	1.0 (5)	3.2 (17)	21.3 (47)	0 (0)	3.6 (4)	3 (15)	0 (0)	14.7 (10)
**Forward translocation**	0 (N/A)	0 (0)	0 (0)	0 (N/A)	0 (0)	0 (0)	0 (N/A)	0 (0)	0 (0)
**Reciprocal translocation**	0 (N/A)	0 (0)	0 (0)	0 (N/A)	0 (0)	0 (0)	0 (N/A)	0 (0)	0 (0)
**Indels at EG**	0 (N/A)	37.4 (195)	44.8 (222)	0 (N/A)	43.3 (52)	19.1 (21)	0 (N/A)	69.7 (235)	41.2 (28)
**LOH + homozygous target edit**	0.7 (4)	3.5 (18)	3.7 (20)	0 (N/A)	0 (N/A)	0 (N/A)	0 (N/A)	0 (N/A)	0 (N/A)
**OT cutting**	0 (0)	21.3 (111)	2.8 (15)	0 (N/A)	8.3 (10)	0 (0)	0 (N/A)	53.7 (181)	1.8 (1)
**Total clones**	100 (599)	100 (522)	100 (538)	100 (221)	100 (120)	100 (110)	100 (505)	100 (337)	100 (68)

Abbreviations: WT = wild-type, LOH = loss-of-heterozygosity, OT = off-target, EG = essential gene

Table S5: ***In silico*–predicted off-target sites for sgRNAs with ≤2 mismatches**.

sgRNA ID	Gene	Chromosome	Target sequence (5' to 3')	OT site #1 (5' to 3')	OT Chromosome	OT Position	OT Gene	Notes on gene
g2	*PPP1R12C* (*AAVS1*)	19	ACCCCACAGTGGGGCCACTAGGG	N/A	N/A	N/A	N/A	N/A
g3	*RAN*	12	AACATCCCCATTGTGTTGTGTGG	AACATCtCCATcGTGTTGTGTGG	4	173634016-173634036	*RANP6*	*RAN* pseudogene
g6	*SF3B1*	2	TGGATGAGCAGCAGAAAGTTCGG	TGGATGAGgAcCAGAAAGTTCGG	11	27658330 – 27658350	*BDNF-AS*	lncRNA

Shown are the predicted off-target sites for each sgRNA, limited to genomic loci containing two or fewer base-pair mismatches across the protospacer and PAM sequence relative to the intended on-target site (genome build: *[hg38]*). Mismatches between targeted sequence and off-target (OT) site #1 shown in lowercase.
